# Identification of differentially expressed genes in female *Drosophila antonietae* and *Drosophila meridionalis* in response to host cactus odor

**DOI:** 10.1186/s12862-014-0191-2

**Published:** 2014-09-02

**Authors:** Camila M Borgonove, Carla B Cavallari, Mateus H Santos, Rafaela Rossetti, Klaus Hartfelder, Maura H Manfrin

**Affiliations:** Departamento de Biologia, Faculdade de Filosofia Ciências e Letras de Ribeirão Preto, Universidade de São Paulo, Av. Bandeirantes 3900, Ribeirão Preto, 14049-900 Brazil; Departamento de Genética, Faculdade de Medicina de Ribeirão Preto, Universidade de São Paulo, Av. Bandeirantes 3900, Ribeirão Preto, 14049-900 Brazil; Departamento de Biologia Celular e Molecular de Bioagentes Patogênicos, Faculdade de Medicina de Ribeirão Preto, Universidade de São Paulo, Av. Bandeirantes 3900, Ribeirão Preto, 14049-900 Brazil

**Keywords:** *Drosophila*-cactus interaction, Differential gene expression, Suppression subtractive hybridization, Adaptation

## Abstract

**Background:**

Studies of insect-plant interactions have provided critical insights into the ecology and evolution of adaptive processes within and among species. Cactophilic *Drosophila* species have received much attention because larval development occurs in the necrotic tissues of cacti, and both larvae and adults feed on these tissues. Such *Drosophila*-cactus interactions include effects of the host plant on the physiology and behavior of the flies, especially so their nutritional status, mating condition and reproduction. The aim of this work was to compare the transcriptional responses of two species, *Drosophila antonietae* and *Drosophila meridionalis,* and identify genes potentially related to responses to odors released by their host cactus, *Cereus hildmannianus*. The two fly species are sympatric in most of their populations and use this same host cactus in nature.

**Results:**

We obtained 47 unique sequences (USs) for *D. antonietae* in a suppression subtractive hybridization screen, 30 of these USs had matches with genes predicted for other *Drosophila* species. For *D. meridionalis* we obtained 81 USs, 46 of which were orthologous with genes from other *Drosophila* species. Functional information (Gene Ontology) revealed that these differentially expressed genes are related to metabolic processes, detoxification mechanisms, signaling, response to stimuli, and reproduction. The expression of 13 genes from *D. meridionalis* and 12 from *D. antonietae* were further analyzed by quantitative real time-PCR, showing that four genes were significantly overexpressed in *D. antonietae* and six in *D. meridionalis*.

**Conclusions:**

Our results revealed the differential expression of genes related to responses to odor stimuli by a cactus, in two associated fly species. Although the majority of activated genes were similar between the two species, we also observed that certain metabolic pathways were specifically activated, especially those related to signaling pathways and detoxification mechanisms. The activation of these genes may reflect different metabolic pathways used by these flies in their interaction with this host cactus. Our findings provide insight into how the use of *C. hildmannianus* may have arisen independently in the two fly species, through genetic differentiation in metabolic pathways to effectively explore this cactus as a host.

**Electronic supplementary material:**

The online version of this article (doi:10.1186/s12862-014-0191-2) contains supplementary material, which is available to authorized users.

## Background

Interactions between insects and their host plants are highly diverse and are an important area of study in evolutionary ecology [1-3]. Examples of these interactions include the effects of host plants on the physiology and behavior of insects, which in turn develop strategies to improve their nutritional status, mating conditions and reproductive success [4,5]. Interest into whether and how differentially expressed genes could be involved in this adaptive process has been a driver for an increasing number of studies aiming to understand the ecological and evolutionary mechanisms underlying these insect-plant interactions [6-9]. The extent and nature of these interactions can now be examined using hypothesis-driven transcriptome analyses based on an ecological perspective [10].

The substantial amount of publically available genomic information on drosophilids, including in-depth data on their evolutionary history and ecological niches, facilitates the study of these questions. Among the drosophilids, the cactophilic species belonging to the Repleta group breed and feed on cactus necrotic tissue [11]. The vast majority of these species are saprophytic, feeding as larvae and adults mainly on yeasts and bacteria growing in decaying tissue of cacti. While yeasts are a major source of nutrients for these drosophilids, these flies are then also exposed to xenobiotic compounds present on the host cactus [12]. Thus, cues for finding the appropriate cactus and necrotic tissue by drosophilids may be an important determinant for their feeding strategies and, ultimately, their reproductive fitness.

For many cactophilic *Drosophila* species the chemical composition of their cactus hosts is one of the major determinants of host-plant specificity [13,14], because of the presence of potentially toxic compounds, such as alkaloids, medium-chain fatty acids, sterol diols, and triterpene glycosides, especially so in columnar cacti [15]. The chemical composition of necrotic cactus tissues is largely dependent on the yeast and bacterial communities present, which metabolize these compounds and produce volatiles that are important cues for the flies to detect possible host plants [16-19].

Among the adaptive genetic changes presented by the *Drosophila* species that use these cacti, primary targets should be detoxification processes to remove or tolerate toxic compounds produced by their host cacti and their microorganism communities [13,20-22]. Furthermore, several studies have demonstrated an influence of these host cacti on fitness-related traits, such as developmental time and viability [23], and on cuticular hydrocarbons [24]. Nonetheless, there is little information on the genetic basis underlying the perception of host cacti necrotic tissue by different *Drosophila* species.

In this study, we used a suppression subtractive hybridization (SSH) approach to compare transcriptional responses of *Drosophila antonietae* and *Drosophila meridionalis* females and identify genes potentially related to responses to odor stimuli released by the columnar cactus *Cereus hildmannianus.* Both species are cactophilic and belong to the Repleta group. Within this group, *Drosophila meridionalis* belongs to the meridiana complex, a monophyletic basal subdivision of the Repleta group, and *Drosophila antonieta* belongs to the buzzatii complex, which is a monophyletic derived subdivision [25]. The two species have the same geographic range, which includes the core Missiones region (Paraná-Paraguay basin river), the South Athantic Seasonal Forest and the eastern part of the Chaco Biome in South America [26-29] and are sympatric in most of their populations. As these species belong to different lineages of the Repleta group and use the same host plant, an interesting question is to understand the genetic pathways and their evolution involved in the exploration of *Cereus hildmaniannus* as a host, which is considered a relevant factor in the diversification of the group [25,30].

Our study revealed the differential expression of a set of genes related to metabolic processes, detoxification mechanisms, signaling, responses to stimuli, and reproduction. We found that a portion of these genes are similar among the two species, while others are different, and we discuss possible functional roles of these genes in the interaction between *Drosophila* flies and their host cactus.

## Materials and methods

### Fly stocks

Both species of cactophilic drosophilids, *D. antonietae* (buzzatii cluster) and *D. meridionalis* (meridiana complex), were maintained in the Laboratory of Evolutionary Genetics of the University of São Paulo, Ribeirão Preto, Brazil. These flies were obtained by collecting decaying cladodes of the cactus *Cereus hildmannianus* from the Experimental Station at Sertãozinho of the Institute of Animal Science and Pastures (IZ), São Paulo, Brazil. The cladodes were placed in a terrarium to allow the flies to emerge. These were then collected, identified and transferred to glass bottles containing culture medium (Additional file 1). From these initial cultures, single females were placed into individual vials to establish isofemale lines. Species identification was refined by analysis of the genitalia (aedeagus) of the male progeny [31], and 10 isofemale lines from each species were mixed to establish the test population used in this study. Populations of both species were reared under identical conditions for 14 generations before the odor exposure tests were conducted.

### Experimental design

**Figure 1:**
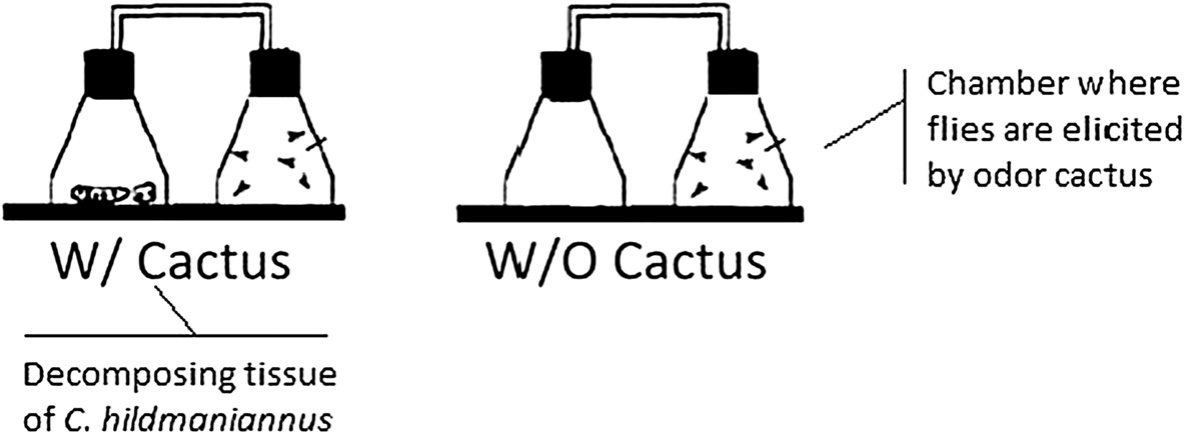
**Schematic representation of the experimental set up.** Flies were placed in 250 ml Erlenmeyer flasks connected by a hose to another Erlenmeyer flask containing decomposing tissue of *C. hildmaniannus* cactus. Flies were prevented by a small pore net from entering the flask containing cactus tissue.

Females of the two fly species that had previously been kept in the presence of conspecific males to enable mating were removed from their culture medium (Additional file 1) 24 h before the start of the experiment and deprived of food (they were placed in the middle of a dish containing 1% agar). From these flies, experimental units were formed consisting of 50 mature female flies (10 to 15 days old) kept in a sealed Erlenmeyer flask. The flasks were maintained in an incubator under controlled conditions (25 ± 1°C, R.H. of 70%) in the dark. Subsequently, the flies of the experimental units were either exposed or not exposed (control) to odor of the cactus *C. hildmannianus* for 6 *hours* (Figure 1). The cactus odor was produced by pieces of fresh cactus and liquid exudate of decaying cladodes from cacti previously collected at the same site as described above. These decaying cladodes had been kept for three days in the laboratory at −20°C, in plastic containers covered by gaze. After a 6 h exposure period to cactus odor, the female flies were immediately frozen in liquid nitrogen and stored at −80°C.

### Analysis of differentially expressed genes (DEGs)

#### mRNA extraction and cDNA synthesis

RNA was extracted from whole fly bodies using TRIzol reagent (Invitrogen) followed by purification with the Pure Link RNA Mini Kit (Invitrogen). The samples were treated with DNase I (Thermo Scientific) to remove genomic DNA from the samples. RNA quantity and purity were estimated by measuring the absorbance at 260 nm and the ratio of the absorbance at 260 and 280 nm, respectively, in a NanoDrop ND-1000 spectrophotometer (NanoDrop Technologies). One microgram of total RNA was used to generate cDNA using the PCR cDNA Synthesis Kit SMARTer (Clontech) according to the manufacturer’s recommendations.

#### Suppressive subtraction protocol

**Figure 2:**
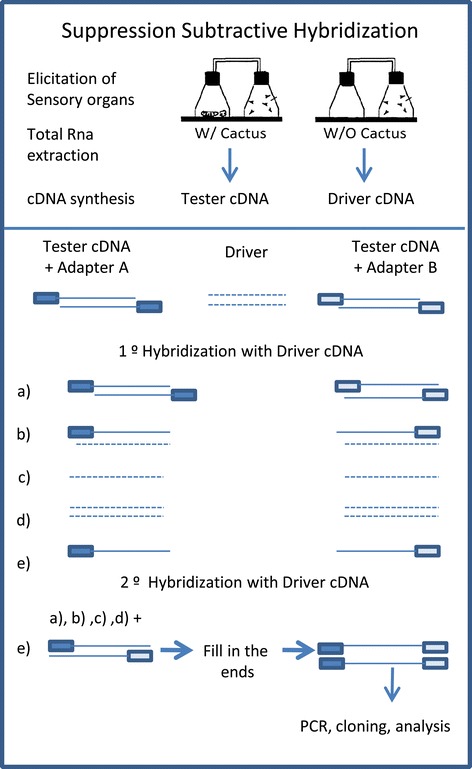
**Schematic diagram of the Suppression Subtractive Hybridization protocol.** Source: Clontech manual modified. Tester cDNA was divided into two aliquots, one for ligation with adapter A and the second with adapter B. Each half was then hybridized with driver cDNA that was not ligated to any adapter. Single-stranded (non-hybridized) sequences of the two independent hybridization steps were then mixed in a second hybridization step generating A-B hynrids. Primers to these adapters were then used for the selective amplification of these hybrids by PCR. This procedure allows for the enrichment of the cDNA population that is overrepresented (differentially represented) in the respective tester cDNA sample.

Differentially expressed genes were identified by means of a suppressive subtraction protocol [32] using the PCR-Select kit (Clontech). This approach permits the enrichment of differentially expressed genes while removing genes that are shared across experimental groups. Briefly, tester cDNA (cDNA from flies that had been exposed to cactus odor) and driver cDNA (cDNA from flies that had not been exposed to cactus odor) was subjected to blunt-end digestion by *Rsa*I restriction enzyme. Tester cDNA was split into two equimolar aliquots to which different PCR primer adapter sequences were ligated (adapter A for one pool of cDNA and adapter B for the other); the primer sequences are described in the PCR-Select kit manual (Clontech). No adapters were ligated to the respective driver cDNA samples. After ligation, the tester cDNAs were mixed with an excess (1:30) of driver cDNA for hybridization. Tester sequences with adapter A that remained single-stranded (did not hybridize to driver cDNA) after the first hybridization round were then used to hybridize with cDNA containing adapter B in the presence of driver cDNA (1:200). Adaptor A-B cDNA hybrids were then selectively amplified by PCR. This procedure allows for an enrichment of the population of cDNA that is specific to (differentially represented in) the respective tester cDNA sample (Figure 2).

PCR products from the subtracted libraries were then ligated into pCR2.1 TOPO TA vector (Invitrogen) for transformation into electrocompetent TOP10 *E. coli* cells (Invitrogen); the transformants were grown on LB-agar media under ampicillin selection. Positive transformants detected by ***β***-galactosidase expression were picked and individually grown in liquid LB-ampicillin medium. For sequencing of the cDNA inserts, the cells were lysed and the plasmids extracted and sequenced using a Big Dye Terminator v1.1 Cycle Sequencing kit (Applied Biosciences) in an automatic sequencer model ABI 3730 XL DNA Analyzer (Applied Biosystems, Foster City, CA).

### Bioinformatics analysis

The sequencing reads were entered into an E-Gene annotation pipeline [33], where they were first filtered to detect and remove reads corresponding to ribosomal and mitochondrial genes. Subsequently, vector sequences were trimmed using the Crossmatch program module implemented in the E-Gene pipeline. Read quality was checked and the reads were assembled using the Phred-Phrap program module. Reads passing these quality checks were next submitted to a CAP3 assembly procedure [34] to obtain unique sequences (USs). These contigs or singlets were then dynamically translated and used in blastx queries against a non-redundant (nr) database (GenBank). The E-value cutoff was set to ≤10^−5^, which is a frequently used value in SSH approaches [35]. ESTs with similarities to genes of known function in other organisms were clustered using Gene Ontology (GO) terms attributed to their respective orthologs in *D. melanogaster* and *D. mojavensis*. Homology and Gene Ontology terms [36], enzyme classification codes (EC), and metabolic pathway analyses of the assembled sequences were retrieved using the BLAST2GO software [37].

### Differential gene expression analysis by quantitative RT-PCR

SSH strategies enrich for differentially expressed genes (DEGs) and, therefore, are not strictly quantitative representations. Differences in expression among identified DEGs must, thus, be validated against independent biological samples. To this end, we performed real-time qRT-PCR analyses with gene-specific primers for a set of selected USs designed by Primer Express Version 3.0 software (Applied Biosystems). The predicted product length varied between 180 bp and 200 bp. An α-tubulin gene recommended for *Drosophila* real time qRT-PCR studies [38] was used as reference gene. In addition, we designed primers to a region of the α-tubulin gene at 84B (CG1913) that is conserved among *Drosophila* species. Primer specificity was tested by conventional PCR. The primer sequences are listed in Additional files 2 and 3.

Fly RNA was extracted with TRIzol followed by purification through a PureLink RNA Mini Kit (Invitrogen) and treated with DNase I (Thermo Scientific) to eliminate possible DNA contaminants. First-strand cDNA was produced from approximately 400 ng of total RNA using a SuperScript II kit (Invitrogen) in a protocol consisting of an incubation at 42°C for 50 min and 70°C for 15 min. The resulting cDNA was diluted five times for use in the real time qRT-PCR assays, which were conducted using SYBR Green PCR Master Mix according to the manufacturer’s protocol (Applied Biosystems). The reaction volume was 14 μl and contained 7 μl of SYBR Green reagent, 1.4 μl of the respective cDNA, 0.7 μl of forward and reverse primers (both at a concentration of 5 μM), and 4.2 μl of nuclease-free water. The amplification reactions were performed in a StepOnePlus system (Applied Biosystems) using the following protocol: 95°C for 20 s followed by 40 cycles of 95°C for 3 s and 60°C for 30 s. Subsequently, dissociation curves were acquired to evaluate amplification product quality by melting peak analysis. The assays were run with the three biological replicates, each as three technical replicates, and with assay controls consisting of non-template samples. The real time qRT-PCR results were analyzed using LinReg software [39]. For statistical analysis, Ct values were used as inputs for randomization tests implemented in the Relative Expression Software Tool (REST) [40], with P ≤ 0.05 considered as statistically significant.

## Results

### Differential expression library characteristics

The SSH libraries from the two *Drosophila* species contained genes enriched in expression in flies exposed to *C. hildmannianus* cactus odor relative to the non-exposed control flies. Among a total of 192 expressed sequence tag (EST) reads for *D. antonietae*, 149 were of high quality (Phred quality ≥20). After assembly, these ESTs resulted in 29 contigs and 18 singlets (47 Unique Sequences, USs). A blastx analysis of these USs showed that 30 out of 47 sequences (63.8%) had significant similarity with genes from the 12 *Drosophila* species with completely sequenced genomes [41], and among these, 24 (80%) were associated with known GO functional categories (Additional file 4).

A total of 336 sequencing reads were obtained in the *D. meridionalis* SSH library, 219 of which were of high quality (Phred quality ≥20). These sequences were assembled into 32 contigs and 49 remaining singlets (81 USs). A total of 46 out of 81 sequences (56.8%) had significant matches with genes from the 12 *Drosophila* species, and for these we could also retrieve GO terms. The remaining 35 genes (43.2%) had no significant matches in GenBank and consequently, also lack GO attributes (Additional file 5). The USs that showed significant matches with genes from *Drosophila* species were submitted to GenBank (JZ544508-JZ544575).

The functional characterization and grouping of DEGs for the two species are shown in the Tables 1 and 2. The results were inferred by using the BLAST2GO program [37]. The majority of the sequences for both *D. antonietae* and *D. meridionalis* were related to the following molecular function GO categories (level 3): ligation (GO:0005488), involving ligation to ions, nucleic acids, proteins, small molecules, cofactors, and transcription factors; catalytic activity (GO:0003824), such transferase, hydrolase,and oxidoreductase; structural molecules (GO:0005198), such as ribosome and nuclear pore constituents; and transporter activity (GO:0005215), including the transport of specific substrates and transmembrane transport (Table 1).

**Table 1:** **Classification of differentially expressed genes (DEGs) according to Gene Ontology (GO) molecular function attributes**

***a). Drosophila antonietae***		
**GO-id**	**GO-term**	**Score**
GO:0000989	Transcription factor binding transcription factor activity	1.0
GO:0036094	Small molecule binding	4.0
GO:0097367	Carbohydrate derivative binding	3.0
GO:0016787	Hydrolase activity	4.0
GO:0030337	DNA polymerase processivity factor activity	1.0
GO:0003735	Structural constituent of ribosome	1.0
GO:0016491	Oxidoreductase activity	2.0
GO:0061134	Peptidase regulator activity	1.0
GO:0043167	Ion binding	8.0
GO:0097159	Organic cyclic compound binding	7.0
GO:0016853	Isomerase activity	1.0
GO:0004857	Enzyme inhibitor activity	1.0
GO:0048037	Cofactor binding	1.0
GO:0016829	Lyase activity	1.0
GO:0005515	Protein binding	3.0
GO:0022892	Substrate-specific transporter activity	1.0
GO:0016740	Transferase activity	5.0
GO:0016874	Ligase activity	3.0
GO:0022857	Transmembrane transporter activity	1.0
GO:0017056	Ttructural constituent of nuclear pore	1.0
GO:1901363	Heterocyclic compound binding	7.0
***b). Drosophila meridionalis***		
**GO-id**	**GO-term**	**Score**
GO:0008144	Drug binding	1.0
GO:0000989	Transcription factor binding transcription factor activity	1.0
GO:0097367	Carbohydrate derivative binding	6.0
GO:0036094	Small molecule binding	7.0
GO:0004871	Signal transducer activity	2.0
GO:0003735	Structural constituent of ribosome	3.0
GO:0016787	Hydrolase activity	4.0
GO:0016491	Oxidoreductase activity	2.0
GO:0061134	Peptidase regulator activity	1.0
GO:0097159	Organic cyclic compound binding	10.0
GO:0043167	Ion binding	11.0
GO:0016853	Isomerase activity	1.0
GO:0004857	Enzyme inhibitor activity	1.0
GO:0048037	Cofactor binding	1.0
GO:0005515	Protein binding	5.0
GO:0003682	Chromatin binding	1.0
GO:0022892	Substrate-specific transporter activity	3.0
GO:0008289	Lipid binding	2.0
GO:0016740	Transferase activity	5.0
GO:0022857	Transmembrane transporter activity	3.0
GO:0017056	Structural constituent of nuclear pore	1.0
GO:1901363	Heterocyclic compound binding	10.0

Female flies were exposed to odor of the cactus *Cereus hildmaniannus*. The analyses were performed using BLAST2GO software [37].

**Table 2:** **Classification of differentially expressed genes (DEGs) according to Gene Ontology (GO) biological process attributes**

***a). Drosophila antonietae***		
**GO-id**	**GO-term**	**Score**
GO: 0002376	Immune system process	1.0
GO: 0044699	Single-organism process	13.0
GO: 0050896	Response to stimulus	5.0
GO: 0009987	Cellular process	20.0
GO: 0008152	Metabolic process	17.0
GO: 0048511	Rhythmic process	1.0
GO: 0032502	Developmental process	7.0
GO: 0071840	Cellular component organization or biogenesis	8.0
GO: 0065007	Biological regulation	8.0
GO: 0000003	Reproduction	4.0
GO: 0040007	Growth	2.0
GO: 0040011	Locomotion	2.0
GO: 0051704	Multi-organism process	1.0
GO: 0051179	Localization	7.0
GO: 0032501	Multicellular organismal process	7.0
GO: 0023052	Signaling	4.0
***b. Drosophila meridionalis***		
**GO-id**	**GO-term**	**Score**
GO: 0044699	Single-organism process	19.0
GO: 0050896	Response to stimulus	8.0
GO: 0008152	Metabolic process	16.0
GO: 0009987	Cellular process	21.0
GO:0071840	Cellular component organization or biogenesis	12.0
GO: 0032502	Developmental process	6.0
GO: 0065007	Biological regulation	9.0
GO: 0000003	Reproduction	3.0
GO: 0040007	Growth	2.0
GO: 0051704	Multi-organism process	1.0
GO: 0051179	Localization	10.0
GO: 0032501	Multicellular organismal process	8.0
GO: 0023052	Signaling	5.0

Female flies were exposed to odor of the cactus *Cereus hildmaniannus*. The analyses were performed using BLAST2GO software [37].

Some terms were species specific. For instance, the *D. meridionalis* SSH library contained sequences related to signaling receptor activity (GO:0038023), signal transducer activity (GO:0004871), drug binding (GO:0008144) and lipid binding (GO:0008289) (Table 1b), and in the *D. antonietae* library we found sequences related to DNA polymerase processivity factor activity (GO:0030337) (Table 1a).

In the biological processes category (set at level 2), genes differentially expressed in response to cactus odor were, for both species, related to the following GO terms: cellular process (GO:0009987), including processes such as cellular recognition, cell cycle, and cellular membrane organization; metabolic processes (GO:0008152), including the metabolism of macromolecules organic, nitrogen, and organophosphate metabolism, and oxidoreduction mechanisms; multicellular organismal process (GO:0032501), including mechanisms related to reproduction and cellular signaling; cellular component organization (GO:0071840); biological regulation processes (GO:0065007); localization (GO:0051179); response to stimulus (GO:0050896); and signaling (GO:0023052) (Table 2).

For *D. meridionalis,* cactus odor-activated genes were also related to neural precursor cell proliferation (GO: 0061351), interspecies interaction (GO: 0044419), establishment of cell polarity (GO: 0030010), and establishment of localization (GO: 0051234) (Additional file 5 and Table 2b). For *D. antonietae*, the DEG list also comprised genes related to cell death (GO: 0016265), immune response (GO: 0002376), response to other organisms (GO: 0051707), and rhythmic behavior (GO:0048511) (Additional file 4 and Table 2a). A statistical analysis (Fisher's exact test) revealed no significant difference for the two species with respect to the number of sequences associated with the GO categories.

Metabolic mapping of the DEGs by KEGG pathway analysis showed that *D. antonietae* females experienced an activation of the following pathways after cactus odor stimulation: inositol phosphate metabolism, lipoic acid metabolism, sphingolipid metabolism, pyrimidine metabolism, and purine metabolism (Additional file 4). In *D. meridionalis*, the activated pathways were glycerophospholipid metabolism; the metabolism of alanine, aspartate, and glutamate; nitrogen metabolism and oxidative phosphorylation (Additional file 5). The phosphatidylinositol signaling system was activated in both species (Additional files 4 and 5).

### Real-time PCR validation

We employed real-time PCR analysis to validate the transcript levels of DEGs revealed in the subtractive libraries of *D. antonietae* and *D. meridionalis* females either exposed or not exposed to *C. hildmannianus* odor. The genes selected for quantitative real time-PCR analysis included those represented by high numbers of ESTs in the respective libraries, and genes with GO attributes related to reproduction, communication, oxidoreduction processes and mechanisms of detoxification. According to previous studies [10], genes involved in these processes are typically activated in *Drosophila* females when they are placed in contact with a host cactus or volatile compounds, such as ethanol, resulting from fermentation by yeast in decomposing cacti. We also included some genes that showed no significant matches to genes of other species, and thus would represent potentially novel genes involved in fly-cactus interaction.

**Figure 3:**
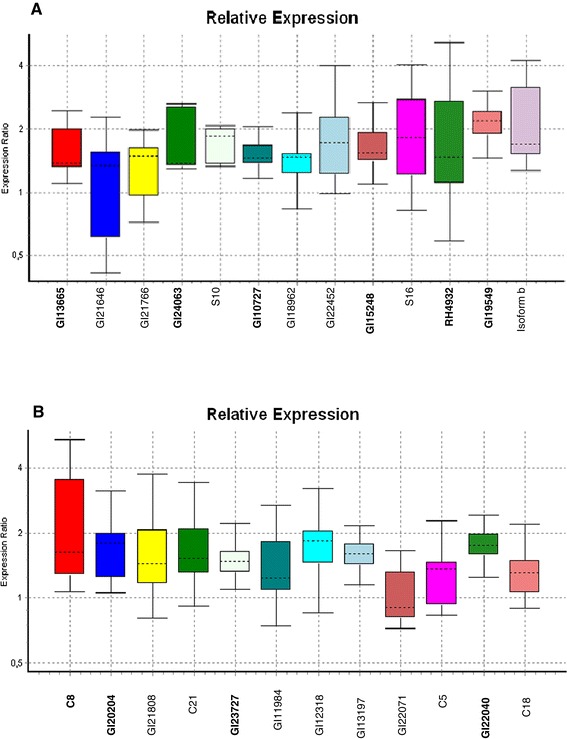
**Relative expression levels in female flies exposed to odor cactus.** Relative expression levels were assessed by quantitative RT-PCR for genes selected from the set of unique sequences of the SSH libraries for odor-exposed females of **(A)** *Drosophila meridionalis* and **(B)** *Drosophila antonietae.* Gene names in bold mark sequences that presented a statistical difference in relative expression (P ≤ 0.05) compared to control (flies not exposed to cactus odor). Statistical analyses were done using the randomization test implemented in REST 2008 [40].

We designed 12 and 13 primer pairs, respectively, for the USs from *D. antonietae* and *D. meridionalis* (Additional files 2 and 3) and used an α-tubulin gene to normalize the expression levels of these genes. In general, all of the genes analyzed by real-time PCR from the two species had a slightly higher expression in flies exposed to cactus odor when compared to non-exposed control flies. For four genes of *D. antonietae* and six of *D. meridionalis* overexpression in odor-exposed flies was denoted as statistically significant (P ≤ 0.05) by randomization tests using REST software (Figure 3) [40], this validating the SSH library results.

The following genes were significantly overexpressed in female *D. meridionalis* flies exposed to cactus odor: Dmoj\GI24063, which is associated with biological reproduction of multicellular organisms; actin-binding protein ipp-like (GI10727), which is involved in the cytoplasmic sequestering of transcription factors; serine threonine-protein kinase 38 (GI13665), which is associated with protein phosphorylation and intracellular protein kinase cascades and the phosphatidylinositol signaling system; alanine aminotransferase (GI15248), which is involved in the metabolism of alanine, aspartate, and glutamate; RH49324p, which has unknown functions; and fk506 binding protein (GI19549), which is involved in protein folding (Figure 3a).

The following genes were found to be significantly overexpressed in *D. antonietae*: trna-dihydrouridine synthase (GI20204), a gene related to oxidoreduction; an f-box and wd-40 domain-containing protein (GI23727) involved in the regulation of signaling mechanisms, olfactory learning, and the ubiquitination of proteins; and a tnf receptor-associated factor (GI22040) related to defense responses, the regulation of the JNK cascade, Toll signaling pathway, receptor activity, protein ubiquitination; and C8 representing a no-match sequence (Figure 3b).

## Discussion

By using an SSH approach we were able to identify genes overexpressed in *D. antonieta*e and *D. meridionalis* females that were exposed to the odor of their host cactus *C. hildmannianus*. The majority of these genes showed significant similaritywith genes of *D. melanogaster*, but several had no known function associated with their sequence (Additional files 4 and 5).

A GO analysis revealed that the known-function genes can be grouped into several categories, including metabolic processes, signal transduction, response to stimulus, and reproduction. Previous studies on the cactophilic species *D. mojavensis* had also implicated the activation of genes belonging to these functional categories in flies reared on their native host cactus, as well as on chemically distinct host tissue [10,20,42].

The metabolic process category encompasses genes involved in the metabolism of carbohydrates, lipids, proteins, and amino acids (Table 2, Additional files 4 and 5). Interestingly, the activation of genes belonging to specific metabolic pathways was detected in each of the species studied herein. For example, genes related to the metabolism of inositol phosphate, lipoic acid, sphingolipids, and purines and pyrimidines were detected in *D. antonietae*, while genes associated with the metabolism of glycerophospholipids, alanine, aspartate, and glutamate were revealed in *D. meridionalis*. The activation of these genes could reflect differences in metabolic strategies employed by the two fly species to survive, develop, and reproduce on their host cactus *C. hildmannianus*.

Genes involved in catalytic activity were up-regulated in response to cactus odor in both species (Table 1). This category included genes encoding hydrolases, transferases, and oxidoreductases. These enzymes are directly linked to the metabolism of xenobiotic compounds typically present in cacti, and previous studies revealed the activation of members of these gene families, including glutathione S-transferases (Gst), cytochrome P450, and UDT-glucuronosyltransferase (UGT), in the detoxification of compounds present in necrotic cactus tissue [10,20]. In our study, a gene belonging to the Gst family was up-regulated in *D. meridionalis* females in response to cactus odor (Additional file 5). These genes are known to be involved in the detoxification of various compounds, particularly hydrophobic organic compounds [43]. In mammals, Gst genes are induced during exposure to carcinogens and in drug-resistance processes [44], and in insects they are associated with insecticide resistance [45]. Surprisingly, only the SSH library for *D. meridionalis,* but not *D. antonietae,* included ESTs representing genes involved in glutathione metabolism. It is possible that this difference resulted from the random selection of clones for sequencing; however, an alternative explanation is that *D. antonietae* employs another mechanism for the detoxification of such compounds. In fact, we found a gene in this species that is linked to lipoic acid metabolism, which is a mechanism employed by some fly species to detoxify insecticides. Lipoic acid acts as an antioxidant by quenching reactive oxygen species that can inflict damage to DNA, proteins and lipids. In *Drosophila melanogaster,* lipoic acid also is effective in counteracting the toxicity of paraquat, a quaternary nitrogen herbicide [46].

Another detoxification strategy used by insects is the excretion of toxic substances. This strategy could explain the activation of genes in both species related to transmembrane transporter activity (Table 1), which is a mechanism that mediates the transport of proteins, lipids, neurotransmitters, and other substrates across cell membranes. For this category, we found a somewhat larger number of genes activated in *D. meridionalis* than in *D antonietae*.

The exploitation of necrotic cactus tissue as a breeding site is an ecological characteristic of most species of the Repleta group. And this required genetic adjustments related to detoxification, as many cacti are rich in chemically complex secondary compounds. The current hypothesis is that the plesiomorphic fly-cactus relationship in the Repleta group is the use of *Opuntia* species as hosts, which are chemically less complex than columnar cacti [25]. Through independent processes, certain *Drosophila* species then evolved to explore chemically more complex columnar cacti that produce toxic compounds, such as alkaloids, medium-chain fatty acids, sterol diols, and triterpene glycosides [15].

Thus, the evolutionarily independent colonization of columnar cacti may have activated different transcriptional pathways in the two species studied here, this depending on the genetic characteristics of their respective ancestral populations. These pathways, when activated by the perception of cactus odors, may represent different metabolic strategies that allow flies to tolerate the toxicity of certain compounds present in tissue of columnar cacti, which, in the present case study, is *C. hilmannianus.*.

In both species, the exposure of female flies to volatile cactus compounds, including ethanol, activated genes related to redox processes (Additional files 4 and 5 and Table 1). Ethanol produces oxidative stress by causing an imbalance in redox reactions and increases the formation of reactive oxygen species (ROS) [47,48]. The increase in ROS levels activates detoxification mechanisms that act as antioxidants and reduce the synthesis of ROS [49]. Glutathione metabolism is among the detoxification mechanisms involved in the response to ethanol and was shown to be activated in *D. meridionalis* in our study. Furthermore, oxidative stress is related to the apoptosis of neurons [50,51]. Interestingly, we found an increased expression of genes related to neurogenesis in both species in this study. At present, we cannot confirm whether this observation indicates a mechanism countering cell death, because most current evidence suggests that neurogenesis does not occur in the brain of adult flies [52] with the exception of a recent study that showed such events by lineage tracing [53].

Genes involved in the ubiquitination of proteins were differentially expressed in *D. antonietae* females exposed to cactus odor (Additional file 4). Oxidative stress increases protein damage and subsequent degradation, and in this process, ubiquitination marks proteins for proteasomal degradation and regulates the protein levels for a number of critical cellular functions, including gene expression [54,55]. In addition, protein ubiquitination plays a role in stress response and in the immune system in *D. mojavensis* [42].

Another important class of genes activated in both species is associated with signaling pathways (Table 2). This category includes genes related to signal transduction mechanisms, such as kinases, phosphatases, and transcription factors. We identified genes related to the Toll signaling pathway, which activates the expression of genes involved in the immune response of *Drosophila* [56]; and the phosphatidylinositol signaling pathway.

Other mechanisms were also specifically activated in each species. In *D. antonietae*, we found evidence for the activation of the Wnt pathway, which plays a central role in several developmental processes, including neurogenesis [57]. This pathway may also be linked to neuronal apoptosis, which can be a result of exposure to ethanol present in volatile compounds produced by cacti [49]. In contrast, the Notch pathway was activated in *D. meridionalis*. This pathway is a node in a conserved signaling mechanism that regulates development [58] and may contribute to plasticity-related processes, including changes in neurite structure [59] and the maintenance of neural stem cells [60]. The Notch pathway is also related to the learning and memory processes [61].

The activation of genes associated with the GO Biological process category “response to stimuli was also common for both species (Table 2), and this category includes G-protein-coupled receptors. These proteins represent a large class of cell-surface molecules that act as stimulus receptors, including receptors for neurotransmitters, hormones, growth factors, odor molecules, and light [62]. In general, these proteins act in response to extracellular signals and activate intracellular signal transduction mechanisms that culminate in the organism's response to the stimulus [63,42].

A noteworthy outcome of the SSH library analyses was the up-regulation of reproduction-related genes in female flies exposed to the odor of necrotic tissue from *C. hildmannianus*. For *D. antonietae* we found four genes involved with reproduction: CG3411, which is involved in oocyte localization in germarium-derived egg chamber formation; CG10262, involved in eggshell chorion formation; CG11989, associated with oogenesis; and CG2512, involved with embryo development ending in egg hatching (Additional file 4). For *D. meridionalis* we found a GI24063 gene associated with multicellular organism reproduction (Additional file 5). These findings are in accordance with those for *D. sechellia* [64], where flies placed in contact with their host cactus showed increased egg production, chorion formation, and oviposition. Thus, olfactory stimuli from necrotic cactus tissues may play an important role in the selection of an appropriate site for oviposition and larval development in *Drosophila* species.

The two fly species investigated herein, *D. antonietae* and *D. meridionalis,* belong to different complexes of the Repleta group. They are sympatric in certain localities along their species range and use the same host cactus as breeding and feeding site. Our data revealed the differential expression of genes related to metabolic processes, signaling pathways, detoxification mechanisms, reproduction, and response to stimuli for both species. While there was an overlap in some of the activated pathways for the two species, there were also specific mechanisms separating the two species. This data provides initial insights into how the use of the *C. hildmannianus* cactus as a host may have arisen by independent adaptation, involving species-specific genetic mechanisms and metabolic and signaling pathway activation in response to cactus odor in the two species.

## Conclusions

In this study, we analyzed the transcriptional response to olfactory stimulation by necrotic *C. hildmannianus* cactus tissue in females of the cactophilic species *D. antonietae* and *D. meridionalis*, which use this cactus as an obligatory breeding site. We found several genes that were differentially expressed in response to cactus odor. The GO and KEGG pathway analysis of these genes showed that the cactus odor activated genes are related to metabolic processes, including the metabolism of carbohydrates, lipids, proteins, and amino acids, as well as to detoxification mechanisms, signaling, response to stimuli, and reproduction. These results provide a basic data set for future investigations into evolutionary mechanisms underlying adaptive diversification processes involved in *Drosophila*-host cactus interactions.

## Additional files

Additional file 1:
**Ingredients for the preparation of culture medium.** This file show the ingredients for the preparation the culture medium used to maintain stocks of *Drosophila* flies. Additional file 2:
**qRT-PCR primer sequences and their respective target genes (when known) for** ***Drosophila antonietae.*** This table show the sequence primer for *D. antonietae* used in qRT-PCR assay. Additional file 3:
**qRT-PCR primer sequences and their respective target genes (when known) for** ***Drosophila meridionalis.*** This table show the sequence primer for *D. meridionalis* used in qRT-PCR assay. Additional file 4:
**ESTs sequenced from the Suppression-Subtractive Hybridization (SSH) library of** ***Drosophila antonietae*** **exposed to odor of the cactus** ***Cereus hildmaniannus*** **.** This table shows the respective orthologs or predicted genes retrieved by blastx searches in GenBank for ESTs sequenced from library of *Drosophila antonietae* exposed to odor of the cactus *Cereus hildmaniannus*. Also included are the Gene Ontology terms and KEGG pathway information. Additional file 5:
**ESTs sequenced from the Suppression-Subtractive Hybridization (SSH) library of** ***Drosophila meridionalis*** **exposed to odor of the cactus** ***Cereus hildmaniannus*** **.** This table shows the respective orthologs or predicted genes retrieved by blastx searches in GenBank for ESTs sequenced from library of *Drosophila meridionalis* exposed to odor of the cactus *Cereus hildmaniannus*. Also included are the Gene Ontology terms and KEGG pathway information.
