# Supplementary material for: Identification of differentially expressed genes in female Drosophila antonietae and Drosophila meridionalis in response to host cactus odor
Source: BMC Evol Biol. 2014 Sep 2;14:191. doi: 10.1186/s12862-014-0191-2 (PMC4161902; doi:10.1186/s12862-014-0191-2)
Supplement: Additional file 1: — Ingredients for the preparation of culture medium. This file show the ingredients for the preparation the culture medium used to maintain stocks of Drosophila flies. [file 12862_2014_191_MOESM1_ESM.docx]

Ingredients for the preparation of culture medium were:

-90g corn grits

-33g of brewer´s yeast

 -1 Tablespoons honey

-9 g agar-

- 1 liters of water
