# Supplementary material for: Identification of differentially expressed genes in female Drosophila antonietae and Drosophila meridionalis in response to host cactus odor
Source: BMC Evol Biol. 2014 Sep 2;14:191. doi: 10.1186/s12862-014-0191-2 (PMC4161902; doi:10.1186/s12862-014-0191-2)
Supplement: Additional file 2: — qRT-PCR primer sequences and their respective target genes (when known) for Drosophila antonietae. This table show the sequence primer for D. antonietae used in qRT-PCR assay. [file 12862_2014_191_MOESM2_ESM.docx]

**Additional file 2**. qRT-PCR primer sequences and their respective target genes (when known) for *Drosophila antonietae*.

| **Gene** | **Primer** | **Primer sequence** |
| --- | --- | --- |
| GI20204 | Forward  Reverse | 5´ GCGCTGTCCGATAGATTGC 3´  5´ GGCTACACTCTTGTCCGCATAGA 3´ |
| GI21808 | Forward  Reverse | 5´ AACCACTCGGGCAGTAGTCATC 3´  5´ GCTCGTTGCCCTCAATCTCA 3´ |
| Unknown | Forward  Reverse | 5´ TGGATGCATTAAGGTTTTACTGTCA 3´  5´ TGTTGGCAGTGCTTGTATTGC 3´ |
| Unknown | Forward  Reverse | 5´GGGCTGCACCATCACCTTT 3´  5´ CACCAAACGTTTCTGCAAAAAA 3´ |
| GI23727 | Forward  Reverse | 5´ TTAGTGGTTCCAGCGATTCCA 3´  5´ GTTTACCATTTCCCCAGAGTTGA 3´ |
| GI22040 | Forward  Reverse | 5´ GCGTAGCCGAATCCTTTGTG 3´  5´ TCGATGGCCGTTGGAAATT 3´ |
| Unknown | Forward  Reverse | 5´ CGACCACTCTTACAACACTAAACAGTT 3´  5´ TTGGACAGGATTTGGATGTGATT 3´ |
| GI11984 | Forward  Reverse | 5´CAATTACTTGCGGTGACATGAAG 3´  5´GTAGCTATAACAATCCGCGGAAA 3´ |
| Unknown | Forward  Reverse | 5´AGCTAGGGTTTCGTTGGGTAAAT 3´  5´ TGAATCTCGTGCACAAATCGA 3´ |
| GI12318 | Forward  Reverse | 5´ GAACGTGCAGCGAAACGTATT 3´  5´ GGCCTCGCAGGCAATG 3´ |
| GI13197 | Forward  Reverse | 5´ CCAACCAGCTGCAAAAAACA 3´  5´ CGGCGGGCAATAGTATACCA 3´ |
| GI22071 | Forward  Reverse | 5´ GAGCGCGCAGTGATCGA 3´  5´GGTGGTAGTCGCATTTGGTATG 3´ |
| α-tubulin | Forward  Reverse | 5´ GGCTTCCTGATCTTCCACTC 3´  5´ CGAACTCCAGCTTGGACTTC 3´ |
