# Supplementary material for: Identification of differentially expressed genes in female Drosophila antonietae and Drosophila meridionalis in response to host cactus odor
Source: BMC Evol Biol. 2014 Sep 2;14:191. doi: 10.1186/s12862-014-0191-2 (PMC4161902; doi:10.1186/s12862-014-0191-2)
Supplement: Additional file 3: — qRT-PCR primer sequences and their respective target genes (when known) for Drosophila meridionalis. This table show the sequence primer for D. meridionalis used in qRT-PCR assay. [file 12862_2014_191_MOESM3_ESM.docx]

**Additional file 3:** qRT-PCR primer sequences with their respective target genes (when known) for *Drosophila meridionalis*.

| **Gene** | **Primer** | **Primer sequence** |
| --- | --- | --- |
| GI21646 | Forward  Reverse | 5´ TCAGAGCAGGAAGCCAATAGC 3´  5´ GCCACACGAACGAGGTCAA 3´ |
| GI21766 | Forward  Reverse | 5´ GAGAATACAACTAAAACTGCGTTGAGA 3´  5´ATTCTTGCGCGTCAATTGG 3´ |
| GI24063 | Forward  Reverse | 5´ CTCTGGGCGGGCTTGAC 3´  5´ ACGTTGCTTGTGCCCAAGA 3´ |
| GI10727 | Forward  Reverse | 5´ TGCTCGATCGCTACTTGTATGTG 3´  5´ CGGAGCTAAGTATGTCCTGGCTAA 3´ |
| GI19549 | Forward  Reverse | 5´ GAAAATGTTAGGTCAGGCAAAGAAA 3´  5´ TTATCTACCAAGGGTGCCTTCTG 3´ |
| GI18962 | Forward  Reverse | 5´ CGCTGCCCGCACTCA 3´  5´ AGGCAGCAAGCCAAGATCA 3´ |
| Isoform b | Forward  Reverse | 5´ GCTGCCAGCGGTGGATAT 3´  5´ CGGCGCCATGGTTGA 3´ |
| GI22452 | Forward  Reverse | 5´ TTGCGGCCATCCAAAGTC 3´  5´ CCTCGGCCATCAGCTTCA 3´ |
| GI15248 | Forward  Reverse | 5´ AAGGAGAAGTCCGAGGTCTTGA 3´  5´ TGCTCAGGGTGTTGTAGACCAA 3´ |
| GI13665 | Forward  Reverse | 5´ CGGGCTTGGAGAAGTCACA 3´  5´ GTTTCGCCTGCGATAAATCAC 3´ |
| Unknown | Forward  Reverse | 5´ GGGAGCGCCTGGCTTAGT 3´  5´ CCATTGGTGGCGGAGCTA 3´ |
| Unknown | Forward  Reverse | 5´ TGCCTGGCACAGTAACTAAAGAATACT 3´  5´ GGCCGAGGTACTCATATTTTCCTT 3´ |
| RH49324p | Forward  Reverse | 5´ CGCCATGCGTCCACAGA 3´  5´ CCCAAGACCAACAAATCTTTGC 3´ |
| α-tubulin | Forward  Reverse | 5´ GGCTTCCTGATCTTCCACTC 3´  5´ CGAACTCCAGCTTGGACTTC 3´ |
