# Supplementary material for: Identification of differentially expressed genes in female Drosophila antonietae and Drosophila meridionalis in response to host cactus odor
Source: BMC Evol Biol. 2014 Sep 2;14:191. doi: 10.1186/s12862-014-0191-2 (PMC4161902; doi:10.1186/s12862-014-0191-2)
Supplement: Additional file 4: — ESTs sequenced from the Suppression-Subtractive Hybridization (SSH) library of Drosophila antonietae exposed to odor of the cactus Cereus hildmaniannus . This table shows the respective orthologs or predicted genes retrieved by blastx searches in GenBank for ESTs sequenced from library of Drosophila antonietae exposed to odor of the cactus Cereus hildmaniannus. Also included are the Gene Ontology terms and KEGG pathway information. [file 12862_2014_191_MOESM4_ESM.docx]

**Additional file 4. ESTs sequenced from the Suppression-Subtractive Hybridization (SSH) library of *Drosophila antonietae* exposed to odor of the cactus *Cereus hildmaniannus*.** The sequence description was obtained by Blast2GO. The respective orthologs or predicted genes retrieved by blastx searches in GenBank are followed by their similarity indices (E-value), Gene Ontology terms and KEGG pathway information.

| Seq description | ID | e-value | GO biological process | GO molecular function | KEGG | GO term |
| --- | --- | --- | --- | --- | --- | --- |
| trna-dihydrouridine synthase | GI20204 CG10436 | 4,1e -26 | oxidation-reduction process; tRNA processing; | flavin adenine dinucleotide binding; nucleic acid binding; tRNA dihydrouridine synthase activity; zinc ion binding; | _ | GO:0050660;GO:0003676  GO:0017150;GO:005514  GO:0008270;GO:0008033 |
| f-box and wd-40 domain protein | GI23727 CG3411 | 3,5e -40 | negative regulation of transforming growth factor beta receptor signaling pathway; olfactory learning; germarium-derived female germ-line cyst encapsulation; regulation of proteolysis; dorsal appendage formation; regulation of centriole replication; ovarian follicle cell stalk formation; locomotor rhythm; regulation of mitosis; negative regulation of Wnt receptor signaling pathway; negative regulation of glial cell proliferation; release of cytoplasmic sequestered NF-kappaB; negative regulation of nurse cell apoptotic process; oocyte localization involved in germarium-derived egg chamber formation; germarium-derived female germ-line cyst formation; border follicle cell migration; negative regulation of smoothened signaling pathway; protein ubiquitination; | phosphoprotein binding; protein dimerization activity; ubiquitin-protein ligase activity; | _ | GO:0030512;GO:0008355  GO:0030708;GO:0030162  GO:0051219;GO:0046843  GO:0046599;GO:0030713  GO:0046983;GO:0019005  GO:0045475;GO:0007088  GO:0030178;GO:0060253  GO:0004842;GO:0045849  GO:0030720;GO:0007298  G0:0045879;GO:0016567 |
| myoinositol monophosphatase | GI21462 CG15743 | 2,7e -62 | inositol biosynthetic process; phosphatidylinositol biosynthetic process; phosphatidylinositol phosphorylation; signal transduction; sulfur compound metabolic process; streptomycin biosynthetic process; | metal ion binding; inositol monophosphate 4-phosphatase activity; 3'(2'),5'-bisphosphate nucleotidase activity; inositol monophosphate 3-phosphatase activity; inositol monophosphate 1-phosphatase activity; | Sulfur metabolism Inositol phosphate metabolism Phosphatdylinositol signaling system Streptomicyn Bioshynthesis | GO:0006790;GO:0019872  GO:0016021;GO:0046872  GO:0052833;GO:0008441  GO:0006021;GO:0052832  GO:0006661;GO:0008934  GO:0046854;GO:0007165;  GO:0005737 |
| thiolester containing protein | GI11285 CG13079 | 1,5e -54 | negative regulation of endopeptidase activity; | endopeptidase inhibitor activity; | _ | GO:0005615;GO:0004866  GO:0010951 |
| proliferating cell nuclear antigen | GI18526 CG10262 | 1,9e -57 | nucleotide-excision repair; neurogenesis; mitotic spindle organization; leading strand elongation; mismatch repair; antimicrobial humoral response; regulation of DNA replication; eggshell chorion gene amplification; | DNA polymerase processivity factor activity; DNA binding; | _ | GO:0006289;GO:0005875  GO:0022008;GO:0030337  GO:0007052;GO:0003677  GO:0006298;GO:0006272  GO:0019730;GO:0043626  GO:0007307;GO:0006275  GO:0005634;GO:0042575 |
| tnf receptor associated factor | GI22040 CG11971 | 1,9e -42 | Toll signaling pathway; phagocytosis, engulfment; instar larval development; dorsal closure; imaginal disc fusion, thorax closure; protein ubiquitination; eye development; positive regulation of apoptotic process; salivary gland cell autophagic cell death; positive regulation of JNK cascade; ventral furrow formation; defense response; asymmetric protein localization involved in cell fate determination; | protein binding; zinc ion binding; ubiquitin-protein ligase activity; receptor activity; | _ | GO:0005515;GO:0001654  GO:0008270;GO:0045179  GO:0008063;GO:0043065  GO:0006911;GO:0035071  GO:0002168;GO:0046330  GO:0007391;GO:0007370  GO:0046529;GO:0004872  GO:0004842;GO:0006952  GO:0016567;GO:0045167 |
| enhancer of variegation 3-9 | GI10181 CG11971 | 2,3e -61 | chromatin maintenance; | nucleic acid binding; zinc ion binding; | _ | GO:0005634;GO:0008270  GO:0070827 |
| GA18797 | GI14139 CG5310 | 1,2e -13 | nucleoside diphosphate phosphorylation; UTP biosynthetic process; GTP biosynthetic process; CTP biosynthetic process; purine nucleobase metabolic process; pyrimidine nucleobase metabolic process; | nucleoside diphosphate kinase activity; ATP binding; | purine metabolism | GO:0006165;GO:0004550  GO:0006228;GO:0005524  GO:0006183;GO:0006241  GO:0006144;GO:0006206 |
| CG10671, isoform a | GI11984 CG10671 | 2,3e -30 | lipid storage; |  |  | GO:0019915;GO:0005789 |
| cationic amino acid transporter | GI14864 CG9413 | 7,6e -14 | leucine import; amino acid transmembrane transport; L-amino acid transport; | L-amino acid transmembrane transporter activity; | _ | GO:0016021;GO:0060356  GO:0015179;GO:0003333  GO:0015807 |
| yata isoform a | GI10423 CG1973 | 1,8e -15 | protein phosphorylation; | ATP binding; protein kinase activity; | _ | GO:0005524;GO:0006468  GO:0004672 |
| CG10778, isoform a | GI14820 CG10778 | 1,7e -27 | phagocytosis, engulfment; | transferase activity, transferring alkyl or aryl (other than methyl) groups; | _ | GO:0006911;GO:0016765 |
| ard 1-like protein | GI12318 CG11989 | 1,5e -40 | neurogenesis; oogenesis; acyl-carrier-protein biosynthetic process; | peptide alpha-N-acetyltransferase activity; | _ | GO:0022008;GO:0004596  GO:0048477;GO:0042967 |
| nucleoporin isoform | GI18159 | 5,7e -29 | nucleocytoplasmic transport; | structural constituent of nuclear pore; | _ | GO:0017056;GO:0005643  GO:0006913 |
| Cip4, isoform a | GI13197 CG15015 | 1,5e -15 | mesoderm development; imaginal disc-derived wing hair organization; signal transduction; negative regulation of synaptic growth at neuromuscular junction; | Rho GTPase binding; | _ | GO:0007498;GO:0005622  GO:0035317;GO:0007165  GO:0045886;GO:0017048 |
| ribosomal protein | GI15807 CG1527 | 2,4e -46 | translation; ribosome biogenesis; | structural constituent of ribosome; | _ | GO:0003735;GO:0005811  GO:0022627;GO:0006412  GO:0042254 |
| lipoate protein ligase | GI22071 CG9804 | 7,5e -53 | cellular protein modification process; lipoate biosynthetic process; acyl-carrier-protein biosynthetic process; | lipoate-protein ligase activity; octanoyltransferase activity; lipoyl(octanoyl) transferase activity; | Lipoic acid metabolism | GO:0016979;GO:0016415  GO:0033819;GO:0006464  GO:0042967 |
| _ | GJ24498 CG3376 | 6,3e -14 | sphingomyelin catabolic process; glycosphingolipid metabolic process; | sphingomyelin phosphodiesterase activity; | Sphingolopid metabolism | GO:0004767;GO:0006685  GO:0006687 |
| alpha-partial | GI23057 CG2512 | 2,4e -15 | positive regulation of growth rate; pronuclear migration; protein polymerization; GTP catabolic process; microtubule-based movement; locomotion; morphogenesis of an epithelium; embryo development ending in birth or egg hatching; | GTPase activity; structural molecule activity; GTP binding; | _ | GO:0040010;GO:0005874  GO:0035046;GO:0051258  GO:0006184;GO:0007018  GO:0040011;GO:0003924  GO:0005198;GO:0002009  GO:0009792;GO:0005525 |
| CG14997, isoform a | GI16584 CG14997 | 1,7e -14 | oxidation-reduction process; | oxidoreductase activity; | _ | GO:0055114;GO:0016491 |
| CG4159, isofrm a  - | GI22173 CG4159 | 9,6e-104 | pseudouridine synthesis; tRNA processing; pyrimidine nucleobase metabolic process; | RNA binding; pseudouridylate synthase activity; pseudouridine synthase activity; | Pyrimidine metabolism | GO:0003723;GO:0001522  GO:0004730;GO:0009982 |
| mediator of rna polymerase ii transcription subunit 22-like | GI15603 CG3034 | 3,1e -34 | regulation of transcription from RNA polymerase II promoter; mitosis; | RNA polymerase II transcription cofactor activity; | _ | GO:0006357;GO:0001104  GO;0007067;GO:0016592 |
| translation initiation factor eif-2b subunit beta like |  | 1,7e -12 | cellular metabolic process; | _ | _ | GO:0044237 |
| zinc carboxypeptidase a 1 partial | GI20107 CG3108 | 6,4e -19 | proteolysis; neurogenesis; | metallocarboxypeptidase activity; zinc ion binding; | _ | GO:0005615;GO:0004181  GO:0006508;GO:0008270  GO:0022008 |
| tetratripeptide repeat protein 9c-like | GI23937 CG17282 | 1,1e-47 | _ | _ | _ |  |
| _ | GI21808 | 5,8e-18 | _ | _ | _ |  |
| cox assembly mitochondrial protein homolog | GI21353 CG17996 | 6,7e-30 | _ | _ | _ |  |
| Atila,isoform b | GI22236 CG6579 | 5,4e-21 | _ | _ | _ |  |
| _ | GI15447 CG9754 | 1,1e-09 | _ | _ | _ |  |
| CG11980,isoform a | GI22581 CG11980 | 4,3e-22 | _ | _ | _ |  |
