# Supplementary material for: Identification of differentially expressed genes in female Drosophila antonietae and Drosophila meridionalis in response to host cactus odor
Source: BMC Evol Biol. 2014 Sep 2;14:191. doi: 10.1186/s12862-014-0191-2 (PMC4161902; doi:10.1186/s12862-014-0191-2)
Supplement: Additional file 5: — ESTs sequenced from the Suppression-Subtractive Hybridization (SSH) library of Drosophila meridionalis exposed to odor of the cactus Cereus hildmaniannus . This table shows the respective orthologs or predicted genes retrieved by blastx searches in GenBank for ESTs sequenced from library of Drosophila meridionalis exposed to odor of the cactus Cereus hildmaniannus. Also included are the Gene Ontology terms and KEGG pathway information. [file 12862_2014_191_MOESM5_ESM.docx]

**Additional file 5**: **ESTs sequenced from the Suppression-Subtractive Hybridization (SSH) library of *Drosophila meridionalis* species exposed to odor of the cactus *Cereus hildmaniannus***. The sequence description was obtained by Blast2GO.The respective orthologs or predicted genes retrieved by blastx searches in GenBank are followed by their similarity indices (E-value), Gene Ontology (GO) terms and KEGG pathway information.

| Seq description | ID | e-value | GO biological process | GO molecular function | KEGG | GO term |
| --- | --- | --- | --- | --- | --- | --- |
| phosphatidylethanolamine-binding protein | CG6180 GI14504 | 7,2e -76 | _ | phosphatidylethanolamine binding; | _ | GO:0008429 |
| pyroglutamyl-peptidase 1 | GI12237 CG32147 | 1,1e -79 | proteolysis; | pyroglutamyl-peptidase activity; | _ | GO:0016920; GO:0006508 |
| CG14997,  Isoform a | GI16584 CG14997 | 1,7e -14 | oxidation-reduction process; | oxidoreductase activity; | _ | GO:0055114; GO:0016491 |
| peroxin 16 | GI13416 Pex16 | 2,7e -13 | spermatocyte division; peroxisome organization; | _ | _ | GO:0048137; GO:0007031 |
| actin-binding protein ipp-like | GI10727 CG9426 | 9,3e -30 | cytoplasmic sequestering of transcription factor; | actin binding; | _ | GO:0042994;GO:0005737; GO:0003779 |
| fk506 binding protein | GI19549 CG5482 | 1,2e -26 | protein folding; | peptidyl-prolyl cis-trans isomerase activity; FK506 binding; | _ | GO:0006457;GO:0003755;  GO:0005528 |
| _ | GI15340 | 1,7e -30 | sodium ion transport; bile acid and bile salt transport; | bile acid:sodium symporter activity; | _ | GO:0016020;GO:0008508;  GO:0006814;GO:0015721 |
| CG10778,  Isoform a | GI14820  CG10778 | 1,6e -27 | phagocytosis, engulfment; | transferase activity, transferring alkyl or aryl (other than methyl) groups; | _ | GO:0006911;GO:00167765 |
| cg10206-pa | GI17742 CG10206 | 1,5e -18 | rRNA processing; | _ | _ | GO:0005730;GO:0006364 |
| translocator protein | GI21766 CG2789 | 1,0e -32 | signal transduction; synaptic transmission; | benzodiazepine receptor activity; | _ | GO:0016021;GO:0005740,  GO:0008503;GO:0007165; GO:0007268 |
| GA17404,  Isoform d | GI24063 | 2,5e -48 | axonogenesis; multicellular organism reproduction; | _ | _ |  |
| drosophila melanogaster partial | GI22925 CG4334 | 9,5e -25 | metal ion transport; transmembrane transport; | metal ion transmembrane transporter activity; | _ | GO:0030001;GO:0016020  ;GO:0046873;GO:0055085 |
| kruppel homolog 1 partial | GI22985 CG6689 | 5,0e -32 | _ | nucleic acid binding; zinc ion binding; | _ | GO:0003676;GO:0005634;  GO:0008270 |
| thiolester containing protein iv | GI112855 | 2,5e- 53 | negative regulation of endopeptidase activity; | endopeptidase inhibitor activity; | _ | GO:0005615;GO:0004866:  GO:0010951 |
| nucleoporin isoform a | GI18159 | 5,7e -29 | nucleocytoplasmic transport; | structural constituent of nuclear pore; | _ | GO:0017056;GO:0005643;  GO:0006913 |
| cdp-diacylglycerol-glycerol-3-phosphate 3-phosphatidyltransferase | GI22216 CG7718 | 3,0e -32 | phospholipid biosynthetic process; glycerolipid metabolic process; | CDP-diacylglycerol-glycerol-3-phosphate 3-phosphatidyltransferase activity; | Glycerophospholipid metabolism | GO:0008444;GO:0008654;  GO:004686 |
| fatty acid binding protein | GI22452 | 1,2e -38 | long-term memory; transport; | fatty acid binding; transporter activity; | _ | GO:0005875;GO:0007616;  GO:0006810 |
| _ | CI19716 CG30344 | 4,0e -52 | response to methotrexate; transmembrane transport; | transporter activity; | _ | GO:0016021;GO:0031427;  GO:0055085;GO:0005215 |
| alanine aminotransferase | GI15248 CG1640 | 4,8e -50 | biosynthetic process; alanine metabolic process; aspartate metabolic process; carbon utilization; | pyridoxal phosphate binding; L-alanine:2-oxoglutarate aminotransferase activity; | alanine, aspartate and glutamate metabolism | GO:0030170;GO:0009058;  GO:0004021;GO:0006522;  GO:0006531;GO:0015976 |
| elongation factor 1 partial | _ | 2,5e -33 | GTP catabolic process; regulation of translational elongation; | translation elongation factor activity; GTPase activity; GTP binding; | _ | GO:0003746;GO:0006184;  GO:0003924; GO:0005525;  GO:0005840; GO:0006448 |
| ribosomal protein l36 | GI11079 Rpl36 | 4,9e -32 | translation; ribosome biogenesis; | structural constituent of ribosome; | _ | GO;0003735;GO:0005811  GO:0022625;GO:0006412  GO:0042254 |
| 60s ribosomal protein l18a | _ | 4,0e -31 | mitotic spindle elongation; centrosome duplication; translation; ribosome biogenesis; | structural constituent of ribosome; | _ | GO:0000022;GO0051298  GO:0003735;GO:0022625  GO:0006412;GO:0042254 |
| ribosomal protein partial | GI12472 Rpl8 | 1,7e -15 | mitotic spindle elongation; centrosome duplication; translation; ribosome biogenesis; | structural constituent of ribosome; | _ | GO:0000022;GO0051298  GO:0003735;GO:0022625  GO:0006412;GO:0042254 |
| GA27251,  Isoform b | _ | 2,9e -16 | interspecies interaction between organisms; endoderm development; | RNA binding; protein binding; | _ | GO:0005730;GO:0003723  GO:0044419;GO:0005515  GO:0007492 |
| cytochrome c oxidase subunit ii | _ | 5,9e -43 | electron transport; mitochondrial electron transport, cytochrome c to oxygen; proton transport; | copper ion binding; electron carrier activity; heme binding; cytochrome-c oxidase activity; | Nitrogen metabolism oxidative  phosphorilation | GO:0005743;GO:0005507;  GO:0009055;GO:0020037;  GO:0016021;GO:0004129;  GO:0006118;GO:0006123;  GO:0015992;GO:0045277 |
| maternal expression at isoform b | GI18279 ME31B | 8,4e -25 | mitotic cell cycle G2/M transition DNA damage checkpoint; cytoplasmic mRNA processing body assembly; | RNA binding; ATP-dependent RNA helicase activity; ATP binding; | _ | GO:0033962;GO:0003723;  GO:0005875;GO;0004004  GO:0000932;GO:0007095  GO:0033962;GO:0005524 |
| tyrosine-protein kinase shark | GI18962 | 1,8e -91 | JNK cascade; protein phosphorylation; dorsal appendage formation; maintenance of epithelial cell apical/basal polarity; dorsal closure, elongation of leading edge cells; apoptotic cell clearance; | ATP binding; non-membrane spanning protein tyrosine kinase activity; | _ | GO:0007254;GO:0006468  GO:0046843;GO:0045199  GO:0005524;GO:0005938  GO:0007394;GO:0004715  GO:0043277 |
| ebi_drome ame: full=f-box-like wd repeat-containing protein ebi | GI21646 Ebi | 1,2e -49 | photoreceptor cell development; wing disc development; compound eye cone cell fate commitment; regulation of proteolysis; positive regulation of Notch signaling pathway; positive regulation of histone deacetylation; regulation of cell cycle; positive regulation of imaginal disc growth; regulation of epidermal growth factor receptor signaling pathway; chaeta development; | GTP binding; repressing transcription factor binding; RNA polymerase II transcription corepressor activity; chromatin binding; | _ | GO:0042461;GO:0005700  GO:0035220;GO:0042676  GO:0030162;GO:0017053  GO:0005525;GO:0045747  GO:0031065;GO:0070491  GO:0051726;GO:0045572  GO:0042058;GO:0001106  GO:0003682;GO:0022416  GO:0000785 |
| serine threonine-protein kinase 38 (ndr2 protein kinase) | GI13665 | 1,3e -42 | dendrite self-avoidance; chaeta development; protein phosphorylation; intracellular protein kinase cascade; regulation of dendrite morphogenesis; imaginal disc-derived wing hair organization; antennal morphogenesis; serine family amino acid metabolic process; | metal ion binding; ATP binding; protein kinase C activity; protein binding; | Phosphatidylinositol signaling system | GO:0070593;GO:0022416  GO:0006468,GO:0007243  GO:0048814;GO:0046872  GO:0035317;GO:0005524  GO:0048800;GO:0004697  GO:0005938;GO:0005515  GO:0005634;GO:0009069 |
| g protein alphai subunit 65a | GI12363 | 3,6e -37 | septate junction assembly; establishment of spindle orientation; GTP catabolic process; asymmetric cell division; ventral cord development; regulation of protein localization; adenylate cyclase-modulating G-protein coupled receptor signaling pathway; cortical actin cytoskeleton organization; establishment of glial blood-brain barrier; asymmetric protein localization involved in cell fate determination; asymmetric neuroblast division; | signal transducer activity; GTP binding; G-protein beta/gamma-subunit complex binding; DNA binding; G-protein coupled receptor binding; GTPase activity; | _ | GO:0004871;GO:0019991  GO:0051294;GO:0005834  GO:0005525;GO:0006184  GO:0008356;GO:0007419  GO:0031683;GO:0032880  GO:0045179;GO:0003677  GO:0001664;GO:0007188  GO:0003924;GO:0030866  GO:0060857;GO:0045167  GO:0055059 |
| nucleolar complex protein | GI23496 CG1234 | 6,5e -27 | fat cell differentiation; | _ | _ | GO:0008150;GO:0045444  GO:0016607;GO:0005730 |
| imune induced molecule 3 | GI19389 CG15068 | 7,0e-08 | defense response; antibacterial humoral;Toll signaling pathway; | _ | _ | GO:0042829;GO:0006961  GO:0008063 |
| proteasome subunit beta type-2 | GI17050 CG17331 | 1,0e-08 | _ | _ | _ |  |
| _ | CJ22669 | 7,6e-10 | _ | _ | _ |  |
| _ | GJ22085 CG15658 | 1,2e-23 | _ | _ | _ |  |
| elongation factor 1 gamma | GI22348 | 1,4e -34 | _ | _ | _ |  |
| _ | GA18797 | 1,2e -13 | _ | _ | _ |  |
| _ | GI21808 | 4,6e -15 | _ | _ | _ |  |
| _ | GI13559 | 4,8e -8 | _ | _ | _ |  |
| _ | GI23927 | 6,1e -42 | _ | _ | _ |  |
| mitochondrial import protein mmp37 | GI23316 CG3331 | 1,4e -47 | _ | _ | _ |  |
| _ | GI18609 | 2,4e -10 | _ | _ | _ |  |
| ribossomal protein S13 | GI23672 | 4,6e -56 | _ | _ | _ |  |
| lsm smlike protein family member | _ | 2,5e -12 | _ | _ |  |  |
| _ | RH49324p | 1,1e -9 | _ | _ | _ |  |
| Lethal (1) GO193, isoform b | GI21656 | 1,0e -26 | _ | _ | _ |  |
